# Supplementary material for: The potential for polyphosphate metabolism in Archaea and anaerobic polyphosphate formation in Methanosarcina mazei
Source: Sci Rep. 2019 Nov 19;9:17101. doi: 10.1038/s41598-019-53168-4 (PMC6864096; doi:10.1038/s41598-019-53168-4)

The potential for polyphosphate metabolism in Archaea and anaerobic polyphosphate formation in *Methanosarcina mazei*.

Fabiana S. Paula[1,2*] and Jason Chin[3], Anna Schnurer[2], Bettina Muller[2], Panagiotis Manesiotis[4], Nicholas Waters[1], Katrina A. Macintosh[3], John P. Quinn[3], Jasmine Connolly[1], Florence Abram[1], John McGrath[3] and Vincent O'Flaherty[1]

[1] Microbiology, School of Natural Sciences and Ryan Institute, National University of Ireland Galway, Galway, Republic of Ireland; [2] Department of Molecular Sciences, Biocenter, Swedish University of Agricultural Sciences, Uppsala, Sweden; [3] School of Biological Sciences and the Institute for Global Food Security, The Queen's University of Belfast, Belfast, UK; [4] School of Chemistry and Chemical Engineering, The Queen's University of Belfast, Belfast, UK.

*Corresponding authors:

Fabiana S. Paula, E-mail: fabianaspaula@gmail.com

Vincent O'Flaherty, E-mail: vincent.oflaherty@nuigalway.ie

Table S1. Query sequences used to retrieve archaeal proteins related to poly-P metabolism.

Protein of interest	Reference sequence NCBI accession number	Reference sequence organism of origin
Polyphosphate Kinase 1	XP_629002.1	*Dictyostelium discoideum* AX4
	WP_000529576.1	*Proteobacteria* spp.
	WP_001078748.1	*Helicobacter pylori*
	WP_001271572.1	*Vibrio cholera*
	WP_004041541.1	*Haloferax volcanii*
Polyphosphate Kinase 2	BAC76403.1	*Acinetobacter johnsonii*
	XP_003080129.1	*Ostreococcus tauri*
	WP_001086042.1	*Vibrio cholera*
	WP_003112634.1	*Pseudomonas* spp.
	WP_004045118.1	*Haloferax volcanii*
Exopolyphosphatase	AAD29107.1	*Pseudomonas aeruginosa*
	NP_012071.1	*Saccharomyces cerevisiae* S288c
	EDU80702.1	*Escherichia coli* O157:H7 str. EC4486
	WP_003120380.1	*Pseudomonas aeruginosa*
	WP_009992804.1	*Sulfolobus solfataricus*

Figure S1. Microscopy images of *M. mazei* cells. A-B) Methylene blue-stained *M. mazei* under brightfield microscopy (A); and the same cells under 405nm excitation (B) - note the dark polyphosphate granules (small dots) visible with methylene blue but not under autofluorescence. C) Unstained *M. mazei* cells excited at 405 nm - note the lack of granules in the autofluorescence. D) DAPI-stained *M. mazi* excited at 405 nm - note the yellow-green polyphosphate granule (* method details).

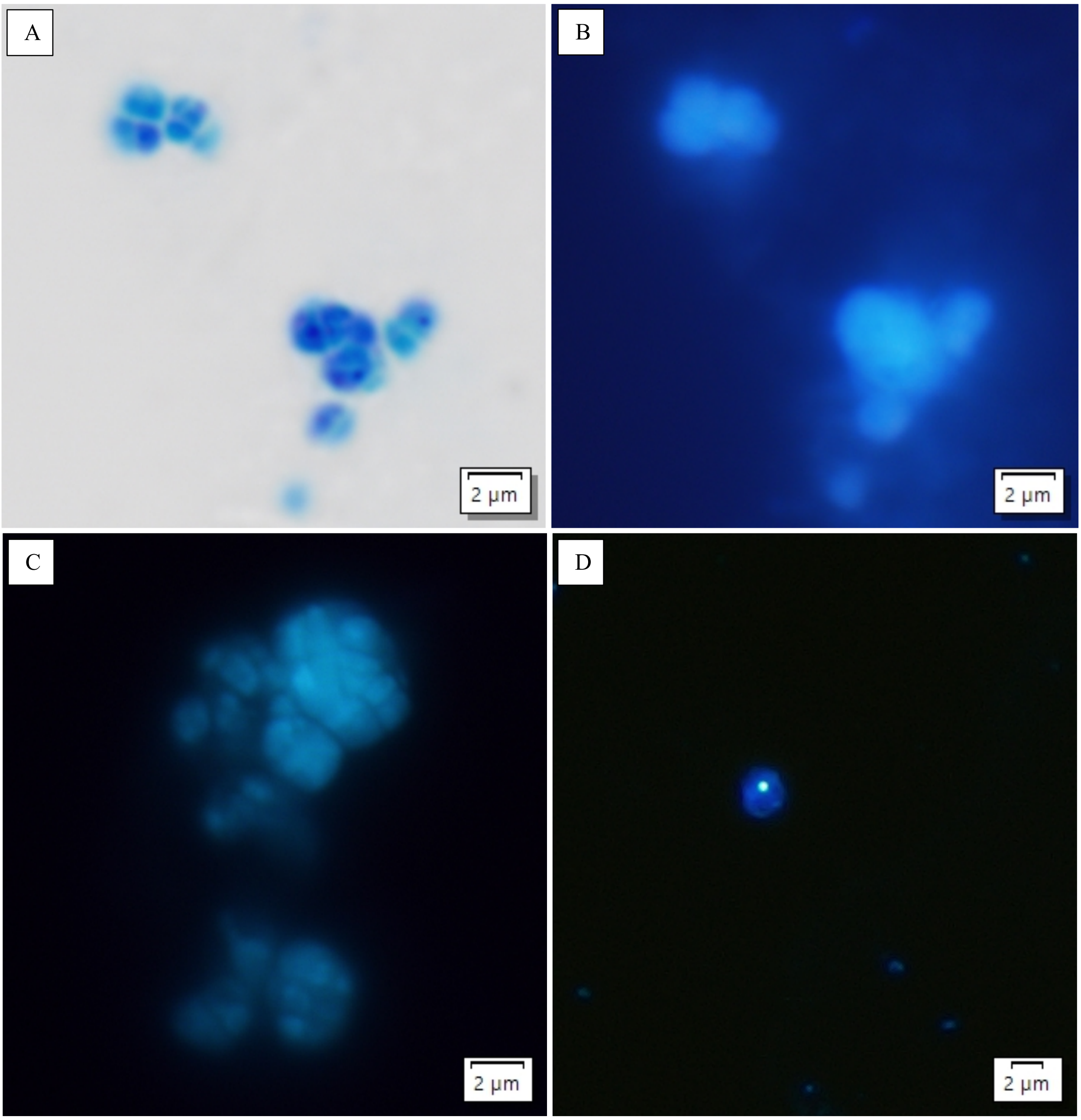

* The cells were grown for 14 days in full medium (methods section). Cell fixation with PFA and DAPI staining procedures were performed as described in the methods section. For methylene blue staining, 5 uL of cell suspension were spread onto a glass slide and air-dried. Cells were stained using methylene blue (Murray et al., 1994) for 45 seconds, washed and air-dried. Images were captured using an Olympus BX63F microscope and an Olympus DP74 camera. Fluorescent images also used a U-FUW filter cube (excitation 340-390 nm, emission 420nm+, mirror 410 nm) and a CoolLED pE-4000 light source with the 405 nm LED.

Murray, R.G.E., Doetsch, R.N., Robinow, C.F. Determinative and cytological light microscopy, (ed. Gerhardt et al.) Methods for General and Molecular Bacteriology. pp28 (American Society for Microbiology, 1994).

Figure S2. Larger versions of Figures 1A, B and C. Cladograms of archaeal homologs of PPK1, PPK2 and PPX proteins. Branch tips represent protein homologs and are coloured according to the order which the sequence belongs to according to the NCBI taxonomic database. Values in the table refer to the number protein homologs retrieved from the database. Symbols outside the cladograms indicate whether (filled symbol) or not (empty symbol) a sequence from an organism with an identical name was found in the PPK1 (red squares), PPK2 (green circles) or PPX (blue triangles) figures for comparison.

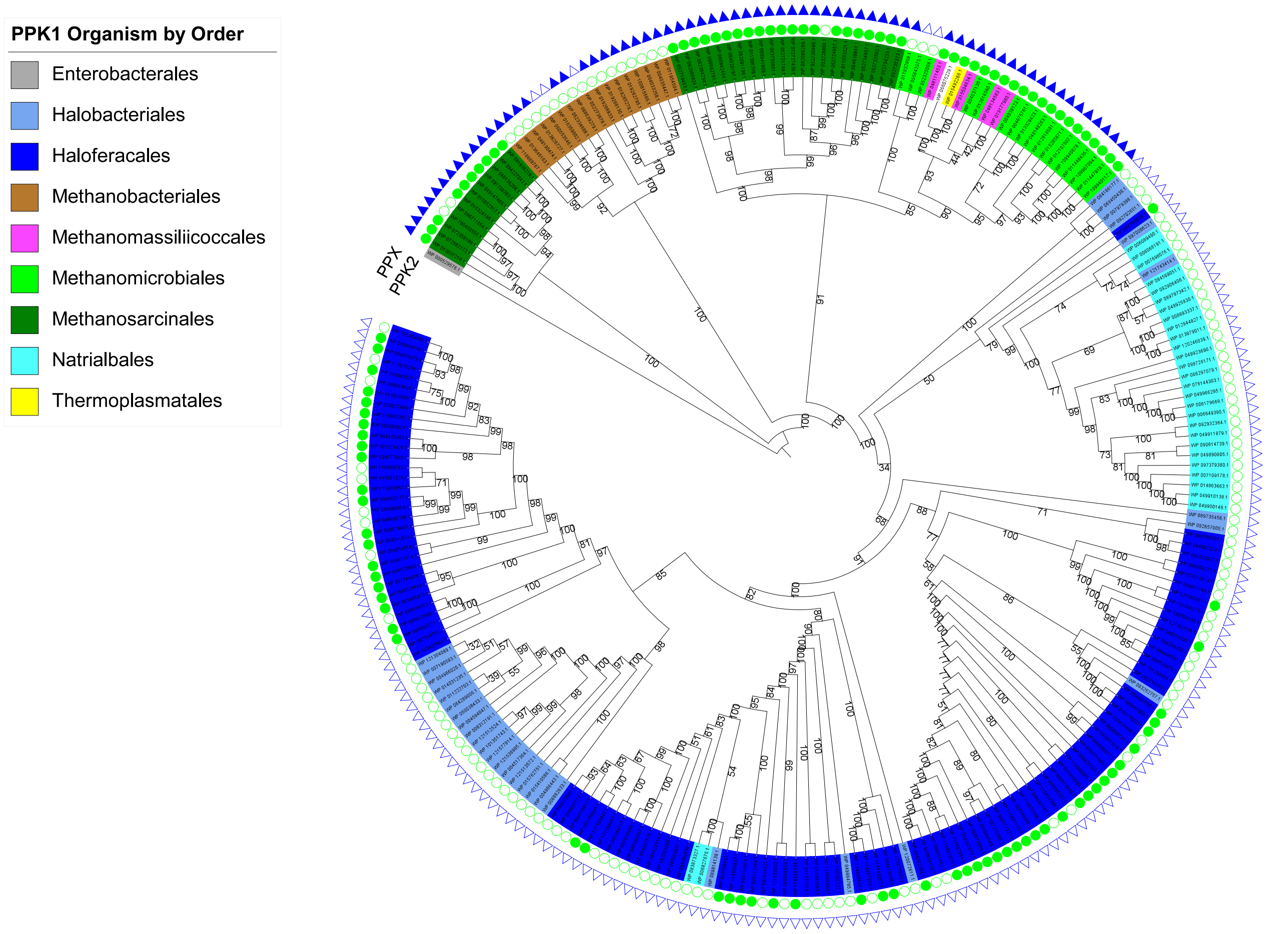

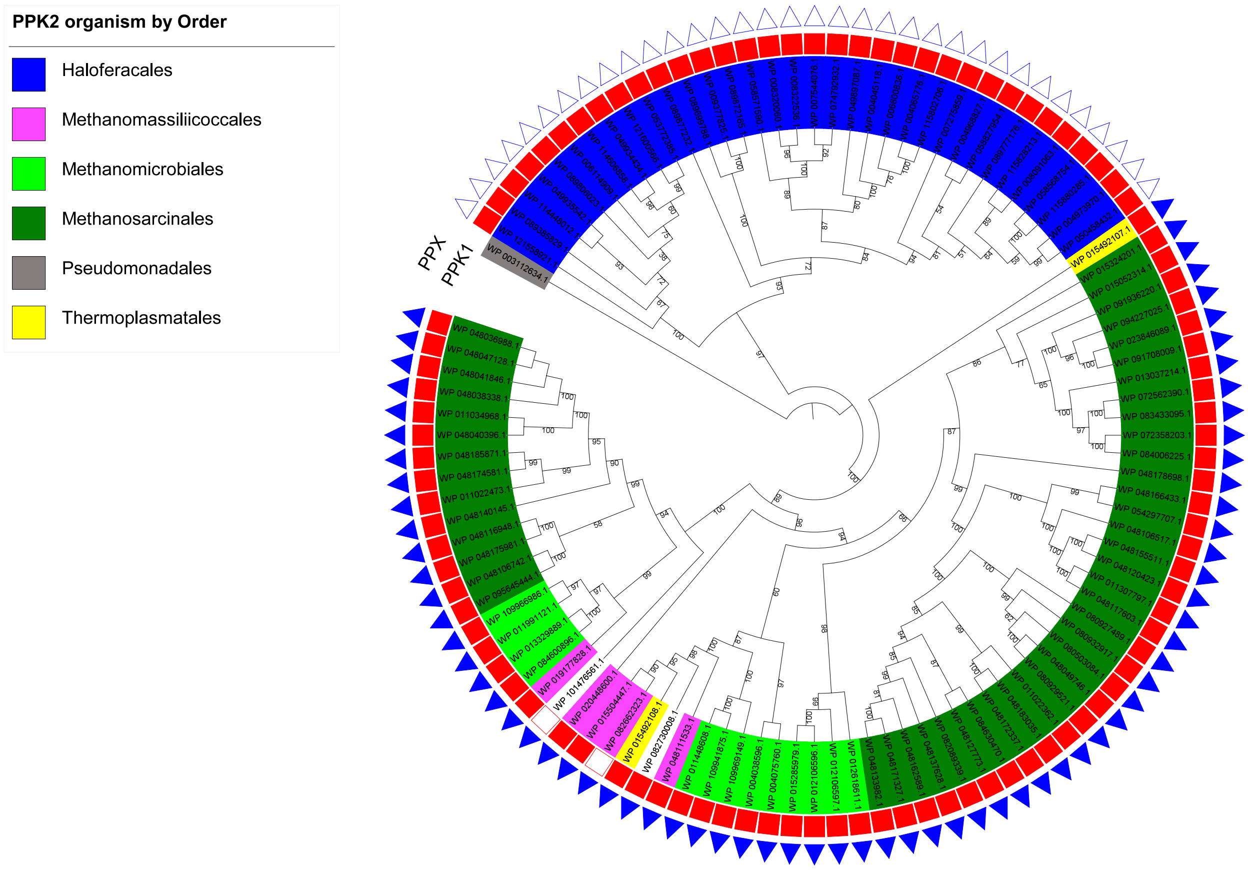
PPK2 organism by Order
Haloferacales
Methanomassiliicoccales
Methanomicrobiales
Methanosarcinales
Pseudomonadales
Thermoplasmatales
PPX
PPK1

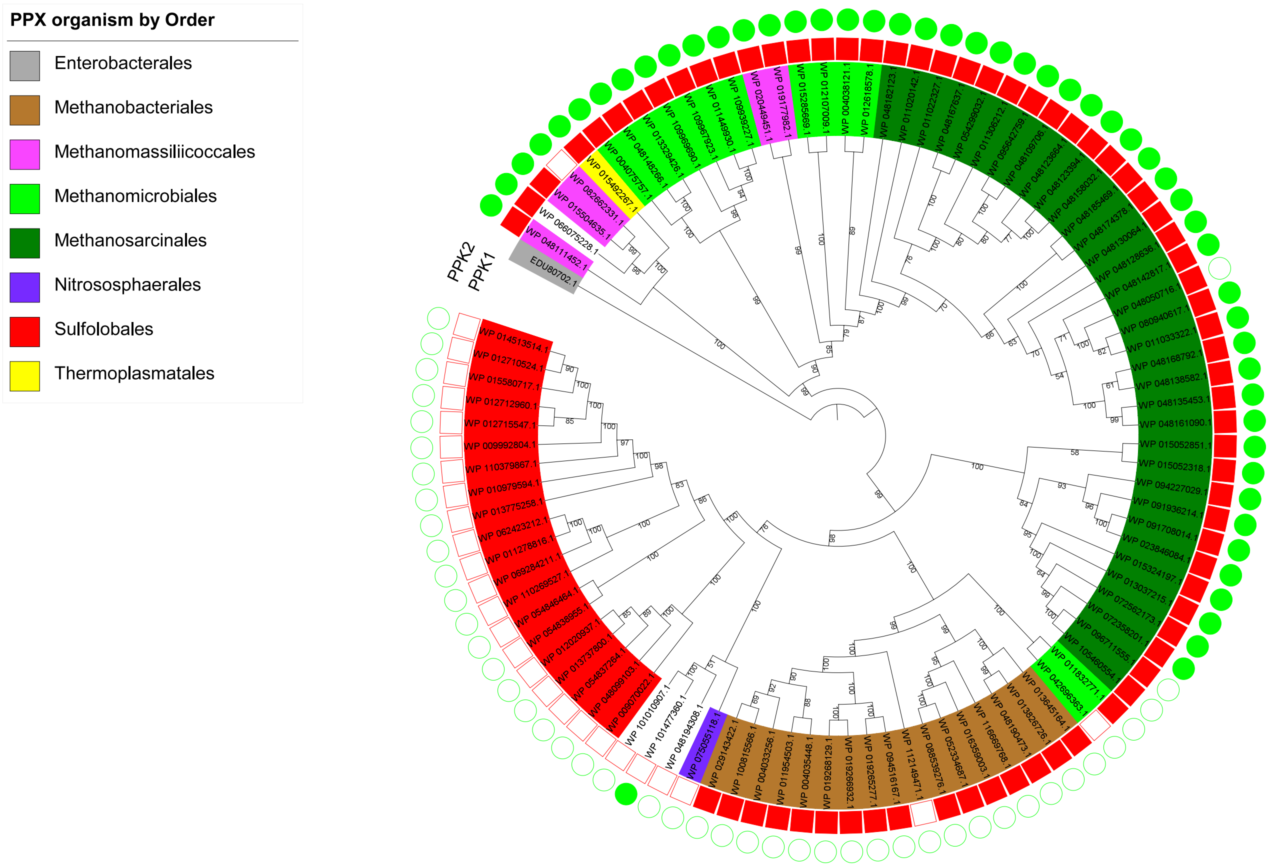
PPX organism by Order
Enterobacterales
Methanobacteriales
Methanomassiliicoccales
Methanomicrobiales
Methanosarcinales
Nitrososphaerales
Sulfolobales
Thermoplasmatales
PPK2
PPK1

Figure S3. Gene ontology (GO) terms for genes A) up- and B) down-regulated in *M. mazei* cells under overplus conditions, in comparison to Pi starved cells. GO terms were retrieved from Uniport (http://www.uniprot.org/taxonomy/). Revigo (http://revigo.irb.hr/) was used to summarise the terms, reduce their redundancy and create the semantic similarity-based charts, which were further formatted using Cytoscape. Node colour intensity indicates the fold-change, i.e. the degree of up- (green) and down-regulation (red) of the respective GO term, as explained in the legend. Highly similar GO terms are linked by edges with width proportional to the semantic similarity. The placement of the nodes was determined by a 'force-directed' layout algorithm that aims to keep the more similar nodes closer together (minor adjustments were applied to facilitate visualization of the GO terms).

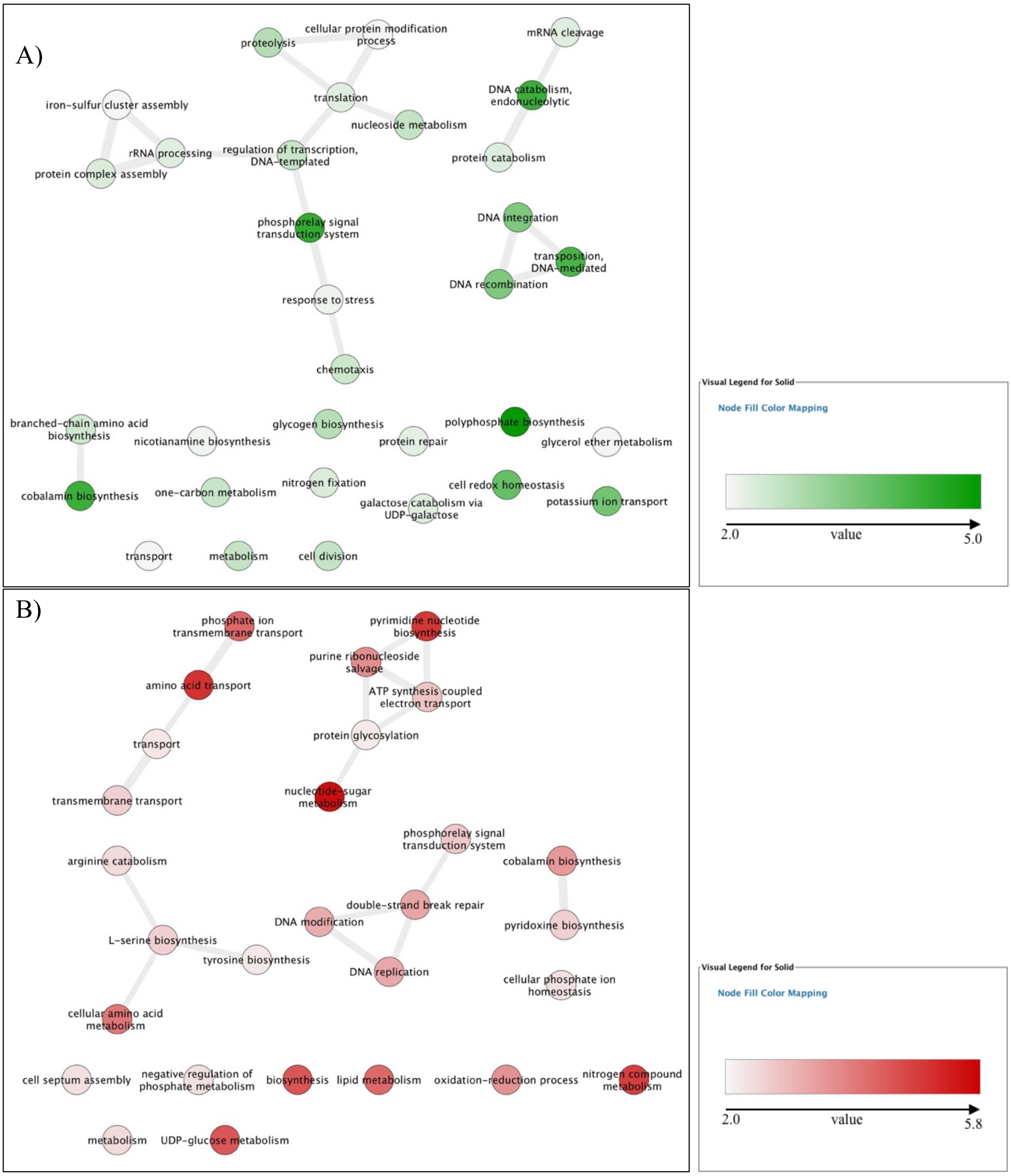

Supplement: Supplementary file 1 — Supplementary Information [file 41598_2019_53168_MOESM1_ESM.pdf]
